# Multimodal Fusion and Transfer Learning for the Detection of Degenerative Parkinsonisms with Dopamine Transporter SPECT Imaging

**DOI:** 10.1007/s10278-025-01831-w

**Published:** 2026-01-20

**Authors:** Valentin Durand de Gevigney, Nicolas Nicastro, Valentina Garibotto, Jérôme Schmid

**Affiliations:** 1 Geneva School of Health Sciences, HES-SO University of Applied Sciences and Arts Western Switzerland, Geneva, Switzerland; 2 Department of Clinical Neurosciences, Geneva University Hospitals, Geneva, Switzerland; 3 Division of Nuclear Medicine and Molecular Imaging, Geneva University Hospitals, Geneva, Switzerland; 4 NIMTLab, Faculty of Medicine, University of Geneva, Geneva, Switzerland; 5 Center for Biomedical Imaging (CIBM), Geneva, Switzerland

**Keywords:** DAT SPECT, Parkinson’s disease, Degenerative parkinsonisms, Multimodality, Transfer learning, Deep learning

## Abstract

Dopamine transporter (DAT) SPECT is a validated biomarker for Parkinson’s disease (PD) and related degenerative parkinsonisms. Interpretation relies on visual assessment supported by striatal image features such as striatal binding ratios (SBRs). Deep learning can aid this process but often underuses complementary data, lacks robustness to heterogeneous inputs, and offers limited interpretability. We built an end-to-end multimodal framework that encodes DAT images and scalar data (patient age and striatal image features) using, respectively, a vision transformer and a multilayer perceptron. A transformer-based fusion module then combined the encoded representations, while tackling possible missing inputs. Interpretability was provided through modality-level attention, spatial attention maps, occlusion analysis, and scalar feature saliency. Performance was evaluated on 664 Parkinson’s Progression Markers Initiative (PPMI) cases and two local datasets A (*N* = 228) and B (*N* = 530) from different devices, including PD, atypical parkinsonisms, and non-degenerative subjects. Transfer learning involved pretraining on two datasets and finetuning on the third. On PPMI, the model reached 97.4% AUROC, 95.5% accuracy, 97.0% sensitivity, and 91.9% specificity, matching state-of-the-art performance. Results were similar on dataset B (98.6% AUROC) but lower on dataset A (92.6% AUROC), likely due to its smaller size and reduced image quality. Explainability analyses showed the model focused on clinically relevant striatal regions and identified key scalar features such as putamen SBR and asymmetry. The fusion module also supported stable predictions despite missing data. Our method efficiently combined multimodal data with heterogeneous datasets and partial multimodal data. Integrated explainability tools showed clinically meaningful attention that is expected to favor its adoption.

## Introduction

The diagnosis of Parkinson’s disease (PD) is mainly clinical, but molecular imaging, for example, dopamine transporter (DAT) SPECT, allows a sensitive evaluation of the integrity of nigrostriatal projections [1–7]. Image analysis is based on the visual assessment of the images supported by semi-quantitative data such as striatal image features, including striatal binding ratios (SBRs). Semi-quantitative data are very helpful in the diagnostic process, notably for the differentiation of parkinsonian syndromes from non-degenerative conditions such as essential tremor (ET) [8, 9].

Machine learning is now widely used for the computer-aided diagnosis of Parkinson’s disease [10, 11]. Initially, machine learning methods such as support vector machines (SVM) and linear regression were trained to predict from low-dimensionality data (e.g., striatal image features) [12–15]. With the emergence of deep learning, researchers evaluated the potential of models such as convolutional neural networks (CNNs) to predict directly from the image, without extracting features from it first [6, 16, 17].

While a visual assessment may not hold enough information to reach the highest specificity [18–20], crossing this visual assessment with clinical and imaging information may improve performance.

Therefore, computer-assisted multimodal methods predicting from multiple data modalities should surpass unimodal methods in the prediction or confirmation of PD. In particular, joint modeling, that is, a way to extract cross-modal information by modeling each modality depending on other modalities, enables models to infer predictions that require information from multiple modalities.

There already exist computer-assisted multimodal methods for predicting or confirming PD, using a variety of data modalities such as speech with handwriting and gait data [2], (^123^I) Ioflupane SPECT (DAT SPECT) images combined with age and sex information [21], or DAT SPECT images with their maximum intensity projection (MIP) and relative extracted features [22].

One significant challenge with multimodal data comes from the heterogeneity that exists between modalities in terms of dimensionality and dynamic range, making their fusion and joint modeling unpractical [23]. Furthermore, deep learning methods are better equipped for jointly modeling heterogeneous multimodal data when one modality has high dimensionality (e.g., images) [23].

Another challenge with multimodal data is the possibility of the absence or incompleteness of one or more modalities, known as incomplete multimodal learning (IML) [24]. Zhan et al. [24] highlighted the importance and difficulty of this challenge and distinguished methods for IML based on the use of internal and external information. These two types are further divided into data-based, feature-based, knowledge transfer–based, graph knowledge enhancement–based, and human-in-the-loop–based methods. The authors stressed that, to be useful in practical cases, multimodal methods should be able to still make robust inferences with partial inputs.

To address these challenges, we developed a multimodal deep learning approach based on transformers, enabling the model to dynamically adjust its attention across modalities depending on content and availability. Deep learning here is key for enabling the simultaneous modeling of images and scalar data, including the age of the patient and striatal image features. Our approach was able to handle heterogeneous data from different datasets with different appearances and data availability in a fully automatic manner. Using the taxonomy from Zhan et al. [24], the proposed method falls into the category of feature-based methods and more specifically attention-based methods.

Two clinical factors shaped our design: limited data availability and trust in the model’s decision. Limited data availability—due to the demands of acquisition, annotation, follow-up, and privacy—makes transfer learning essential for leveraging prior knowledge from existing datasets while minimizing domain shift caused by imaging variability. Limited data availability also translates into partial data (e.g., missing striatal image features), making flexibility in regard to available inputs a desirable feature. To foster clinical trust and usability, we integrated explainability methods enabled by the deep learning framework that offer interpretable insights into model predictions.

The main contributions of our work are as follows:

1. Incomplete multimodal learning: We developed an efficient way to jointly model imaging and scalar data, supporting incomplete data at training and inference stages.
2. Transfer learning: We evaluated the clinical impact of transfer learning in order to leverage prior knowledge from previous heterogeneous datasets.
3. Explainability: We investigated various explainability methods, from intra- and inter-modality perspectives, using attention, occlusion, and saliency techniques.
4. The resulting multimodal approach demonstrated competitive performance across diverse datasets, matching that of state-of-the-art techniques.

## Material and Methods

### Datasets

**Fig. 1:**
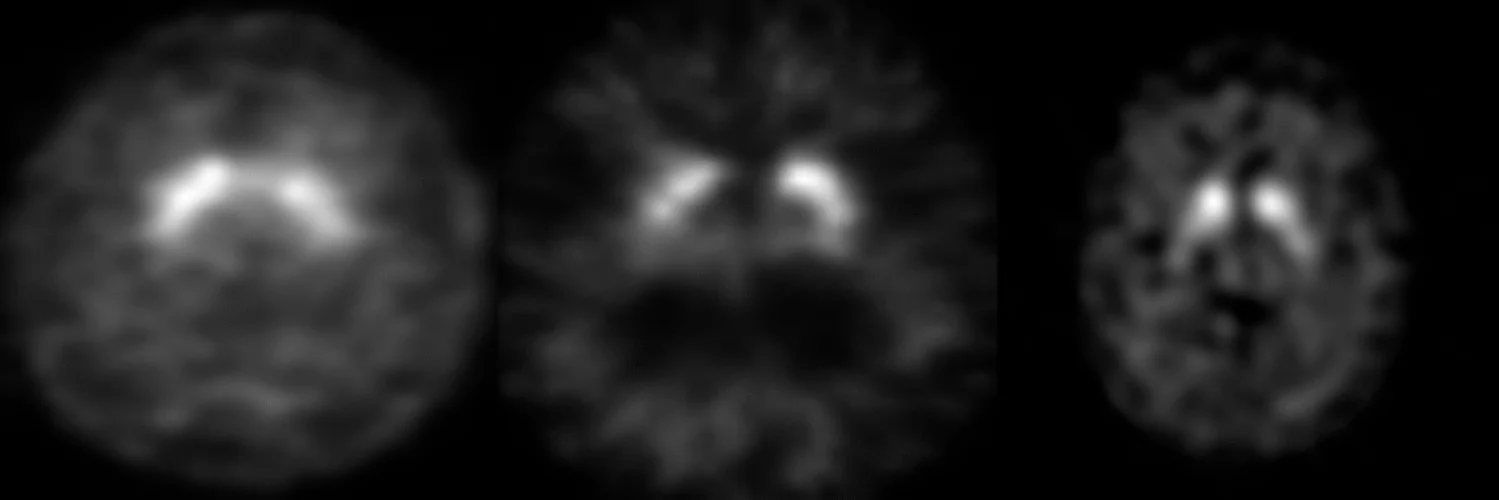
Examples of SPECT images for each dataset. From left to right: PPMI, Local A, Local B datasets. Examples shown are images before any homogenization. The homogenization process is fully automatic and works with the different scales and appearances shown in this figure

To evaluate our methods, we used the public PPMI dataset [25] and two local datasets A and B. In Table 1, we summarize the main characteristics of each dataset, and some image examples are illustrated in Fig. 1.

**Table 1:** Summary of the datasets, including data from PPMI and from local datasets A and B. For the PPMI dataset, prevalence refers to the proportion of PD cases, whereas for local datasets, it denotes the percentage of degenerative parkinsonism cases

Dataset	Count	Prevalence	Mean age ± standard deviation	Female percentage	Image striatal feature count
PPMI	664	70.2%	61 ± 10	35%	4
Local A	228	47.8%	73 ± 11	49%	14
Local B	530	67.7%	70 ± 11	49%	14

#### Data Modalities

**Fig. 2:**
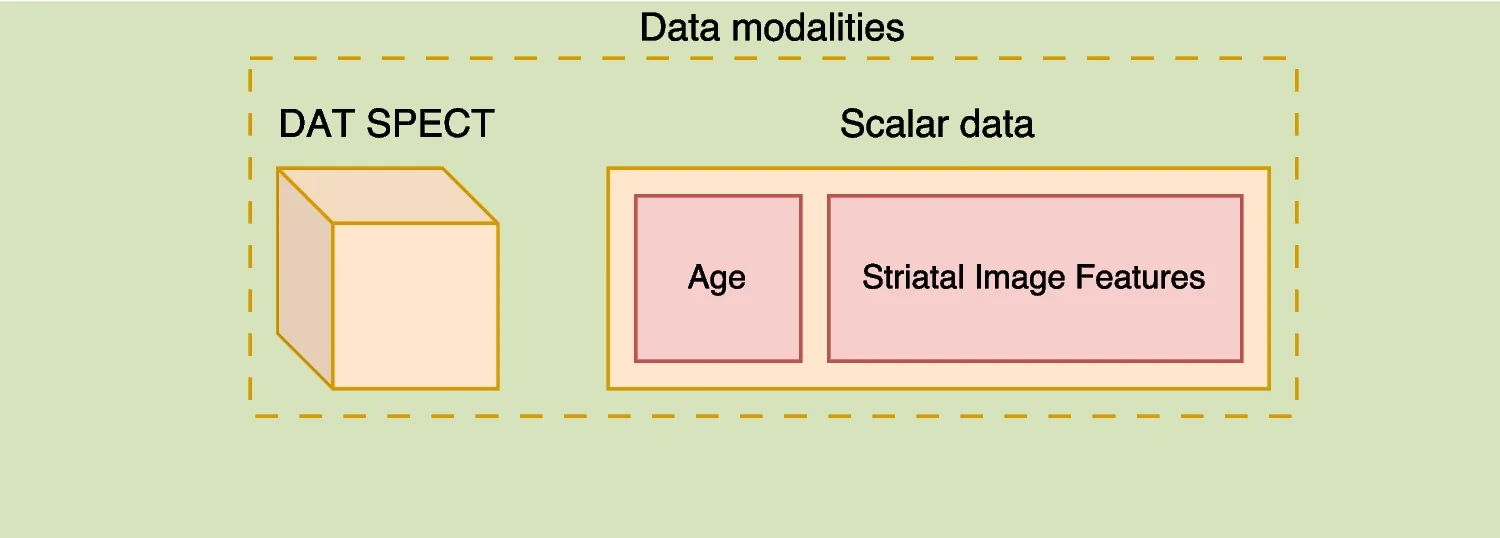
Data modalities used in this work. Scalar data are composed of both the age of the patient and the striatal image features computed from the DAT SPECT image

In this paper, we distinguish two types of data modalities. The first consists of 3D DAT SPECT images and the second of scalar data. In the present context, scalar data are vectors with two distinct feature sets: patient age and striatal image features (such as SBR). These data modalities are illustrated in Fig. 2.

While datasets contained other data modalities, these data modalities were selected because they were present in all datasets—albeit with some heterogeneity—and because their relevance has been well demonstrated. For scalar data, striatal imaging features have been well developed and standardized to support the detection of parkinsonisms [12–15]. Given a significant decline in striatal dopamine levels occurs with age [26], striatal image features can be age-matched to improve detection [27]. Therefore, scalar data vectors were composed of the age of the patient and striatal image features. However, local datasets had additional striatal image features that the PPMI dataset did not have. We detail this difference in the following sections describing the specificities of each individual dataset. Heterogeneity between datasets was notably due to this difference of striatal image features and to the difference in image acquisition and registration detailed in the following sections. It is worth noting that image quality was subjectively different between datasets, and to our best knowledge, no validated objective approach has been proposed to quantitatively assess the quality of DAT SPECT images.

#### Shared Characteristics

In all three datasets, DAT SPECT images were aligned to the Montreal Neurological Institute (MNI) space. PPMI images were already registered to the MNI space [28] and images from our local datasets were registered using DaTQUANT 2.0 (GE Healthcare). The first four striatal image features were the same: four SBR calculated as the left and right caudate and putamen uptakes relative to occipital cortex uptake.

#### PPMI

Images were acquired between 2010 and 2020. The clinical diagnosis was reliably established using a variety of diagnostic techniques (imaging, motor assessments, etc.) [28]. PPMI was considered as a reference dataset, both in terms of overall quality and of how often it is used in the literature to train and evaluate computer-aided diagnosis methods. Acquisition protocols, the image reconstruction, and the method to extract the four SBRs we had access to are detailed in PPMI documentation [28].

#### Local Datasets

We used DaTQUANT 2.0 recompiled for a 64-bit Linux platform for registering images to the MNI space and for computing striatal image features for our local datasets A and B. DaTQUANT generated 10 additional striatal image features: left and right SBR of the whole striatum and anterior putamen and posterior putamen, as well as left and right putamen to caudate uptake ratios and asymmetry values for the caudate and putamen. The asymmetry value for a region R was expressed as $0.5 * |{R}_{\mathrm{L}}-{R}_{\mathrm{R}}|/({R}_{\mathrm{L}} + {R}_{\mathrm{R}})$, where ${R}_{\mathrm{L}}$ and ${R}_{\mathrm{R}}$ denote the mean uptake value in the left and right sides of the region. For each patient, the clinical diagnosis was made using established clinical criteria for degenerative parkinsonisms with the following subtypes: PD [29], corticobasal syndrome (CBS) [30], multiple system atrophy (MSA) [31], and progressive supranuclear palsy (PSP) [32]. More information about the degenerative subtypes present in the local datasets can be found in Table 2.

**Table 2:** Additional information about local datasets, including the degenerative subtypes of Parkinson’s disease (PD), corticobasal syndrome (CBS), multiple system atrophy (MSA), and progressive supranuclear palsy (PSP)

Dataset	Count	Period	Degenerative subtypes			
			PD	MSA	PSP	CBS
Local A	228	2016–2019	86	10	11	2
Local B	530	2003–2017	277	27	30	25

The first local dataset (Local A) included images acquired between 2016 and 2019 using a Siemens Symbia gamma camera, comprising 109 degenerative parkinsonism cases and 119 control cases. Images were acquired on a Siemens Symbia™ T system equipped with low-penetrance high-resolution collimators. The patient’s head was positioned in a dedicated head holder to minimize motion, and acquisition was performed with the following parameters: rotational radius kept between 13 and 15 cm; matrix 128 × 128; 120 projection angles over 360°. Reconstruction was done using Flash3D (OSEM3D with resolution recovery) with 10 iterations and 8 subsets, a triple-energy window method for scatter correction and attenuation correction using low-dose CT.

The second local dataset (Local B) included images acquired between 2003 and 2017 using a Toshiba gamma camera specifically designed for head acquisition, resulting in well-positioned brains in images (Fig. 1). It featured 359 degenerative cases and 171 control cases. A close version of this dataset has been used in previous studies [33, 34]. Data acquisition and reconstruction protocols are thoroughly detailed in the first study [33].

### Data Preprocessing

In this section, we detail the different steps we took to pre-process the data before training, depending on the modality type.

#### DAT SPECT Images

**Fig. 3:**
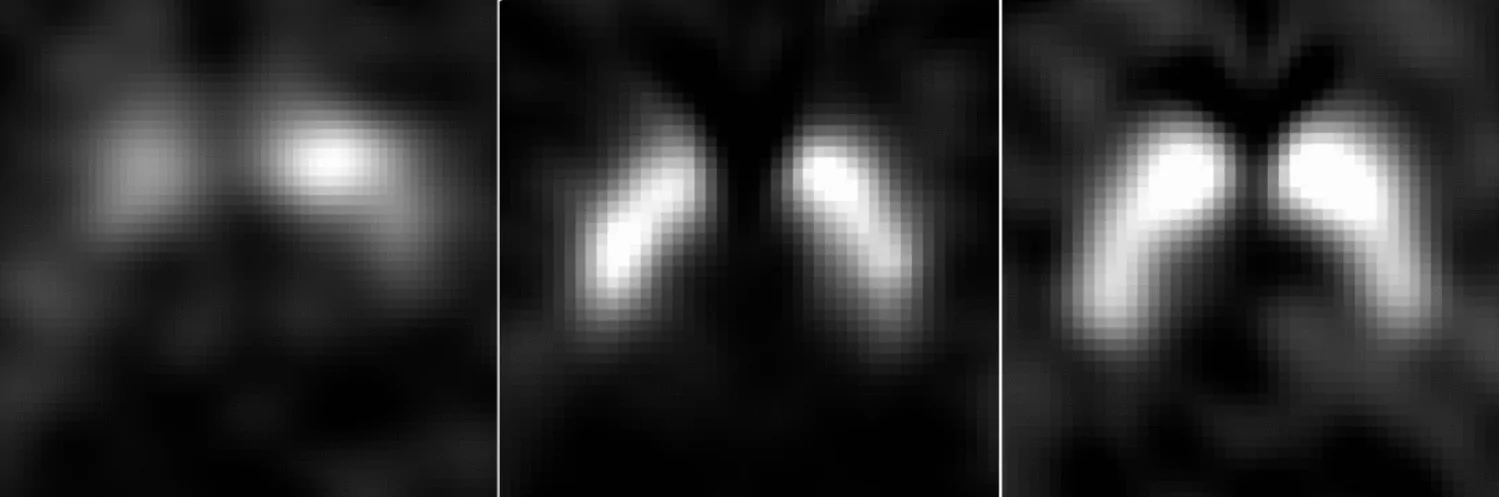
Examples of pre-processed SPECT images. From left to right: PPMI, Local A, Local B

The intensity of each image was scaled between zero and one individually before standardization (also known as zero-mean normalization) using the average intensity and standard deviation of all intensity-scaled images in the dataset. In the case of transfer learning, this intensity standardization was performed independently for each dataset, for example, images in the PPMI dataset were standardized based on the mean and standard deviation of PPMI images, independently of other datasets. Then, we applied a dynamic crop around the striatum. The crop was centered depth-wise on the slice with the highest average intensity and was fixed for other dimensions. The size of the cropping volume was a constant region of 64 × 64 × 32 voxels with a spacing of 1.5 × 1.5 × 2 mm, resulting in a region size of 96 × 96 × 64 mm as depicted in Fig. 3.

#### Scalar Data

Like images, scalar data were standardized individually (each feature was shifted and scaled by the corresponding average and standard deviation values over the dataset). The 10 additional striatal image features missing in the PPMI dataset were replaced by zeros, corresponding to the mean after standardization. To support incomplete multimodal learning, an “availability” vector for the scalar features was computed based on the presence of each individual scalar feature (1.0 for present, 0.0 for missing).

### Model Design

In this work, we did not classify specific degenerative parkinsonisms, but we built a model to distinguish between degenerative and control cases based on the various modalities. In order to take advantage of the two data modalities—DAT SPECT images and scalar data—we built a deep learning multimodal model. When building such a multimodal model, it is necessary to determine the fusion method which will dictate how the different modalities will be combined to arrive at a single prediction.

To fuse multiple modalities, there are three possibilities: early fusion, intermediate (or joint) fusion, and late fusion [35]. Early fusion happens directly in the input space, before the model, while late fusion takes place in the output space, usually aggregating predictions from unimodal sub-models. Intermediate fusion operates at an intermediate stage where modalities have been projected to a shared representation space, before the output space.

Early fusion would have been impractical considering the gap between the dimensionality of the modalities we used. 3D images are too different from scalar data to use early fusion in a practical manner. On the other hand, late fusion does not allow for cross-modal modeling. Put otherwise, late fusion does not allow for inferring predictions that require access to high-level information from multiple modalities at once. Therefore, we chose to use intermediate fusion, which solves the problems posed by early and late fusion mentioned above. Intermediate fusion is also the recommended fusion in medical imaging [35–39]. However, intermediate fusion is not a straightforward process and poses its own challenges.

To perform intermediate fusion, we designed our model the following way: First, a set of unimodal encoders are tasked to project individual modalities to a shared representation space. Then, these representations are fused into a single representation using a transformer encoder. This fused representation is then fed to a final sub-model that yields the model’s prediction—a single logit. As described in Sect. 2.2.2, an availability vector is built to indicate to the model if there are missing scalar features (and if so, which ones). This availability vector requires an encoder of its own, acting as a “third” data modality.

**Fig. 4:**
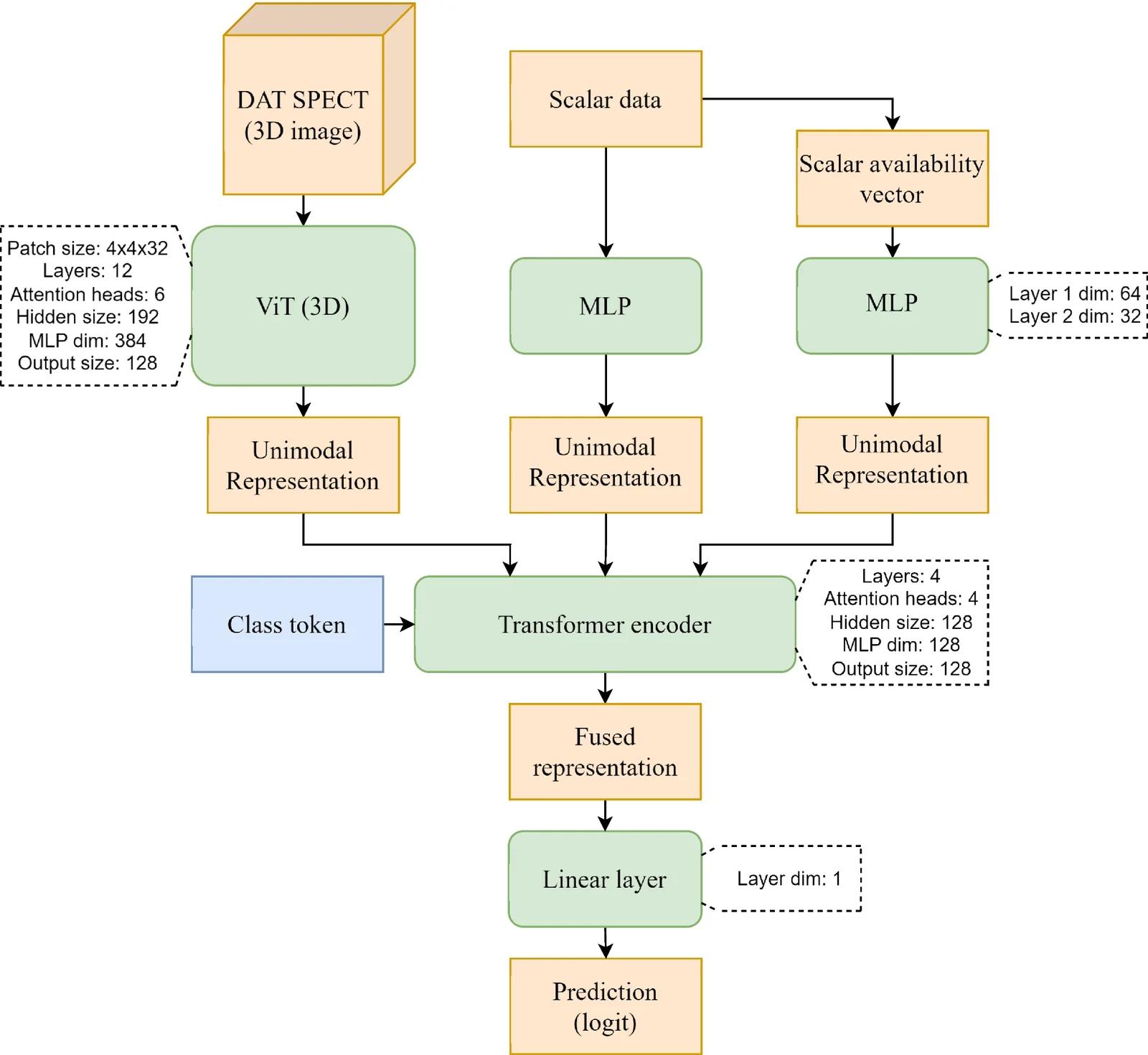
Graph representation of the architecture supporting different modalities by relying on various sub-models to encode and fuse the data. The scalar mask is based on the availability vectors indicating which parts of the scalar content are present and thus valid. A class token inserted at the beginning of the sequence is given to the transformer encoder, whose output is used as the joint representation for making the prediction

In total, our method uses a total of five sub-models. Three encoders—for the image, the scalar data and the availability vector—the transformer encoder performing intermediate fusion and finally a single linear layer projecting the fused representation into the model’s prediction. To encode DAT SPECT images, we used a vision transformer (ViT) [40] adapted for 3D (without its classification layers). To encode scalar data—features and the availability vector—we relied on a simple multi-layer perceptron (MLP). Both transformers (the ViT and the fusion transformer) added a “class token” as well as positional embeddings, as commonly found in the literature [40]. We present a graphical representation of this architecture in Fig. 4.

### Hyperparameters

#### Multimodal Model Hyperparameters

##### Image Encoder

The ViT used 4 × 4 × 32 patches, 12 layers each using 6 heads with a hidden size of 32. The size of the output linear layer of the ViT layers was set to 384. The size of the final output layer was 128. Patches were encoded using a convolutional layer, instead of a linear layer. As with the original transformer and the ViT, we made use of an additional class token. The corresponding output of this class token was used as the final output of the image encoder.

##### Scalar Features Encoder

Scalar features were encoded using a 3-layer MLP. The layers were separated with rectified linear unit (ReLU) activations and used the following hidden sizes in order: 64, 32, and 128.

##### Multimodal Fusion Module

The fusion module was a 4-layer transformer with 4 heads, each with 32 hidden units, and used both a class token and positional embeddings. The output of the fusion module was directly projected to logits with a final linear layer. The size of the input and output tokens was 128.

##### Final Classification Module

The output of the fusion module was then passed to a single linear layer, transforming the fused representation to a single class logit, in turn transformed into a probability with the sigmoid function.

##### Training Hyperparameters

Models were trained over 200 epochs using the AdamS optimizer [41] with a base learning rate of 2e^−4^ and a weight decay of 5e^−4^. Loss function was the binary cross-entropy.

Learning rate was learning warmed-up over 10 epochs from 1e^−5^ to the base learning rate [42]. Afterwards, the learning rate was decayed following a cosine annealing schedule [43], with a decay rate set at 0.9998. The batch size was set to 64.

#### Image and Scalar Model Hyperparameters

Image-only models used the same image encoder hyperparameters as the image encoder from multimodal models. To produce logits, a final linear layer was added after the image encoder. Training hyperparameters were the same as multimodal models.

For scalar data, logistic regression models used a maximum number of iterations of 1000, the norm penalty was the L2 norm, the strength of the inverse regularization was set to 1.0, and we used the limited-memory Broyden–Fletcher–Goldfarb–Shanno (BFGS) optimization algorithm.

### Training and Testing

**Fig. 5:**
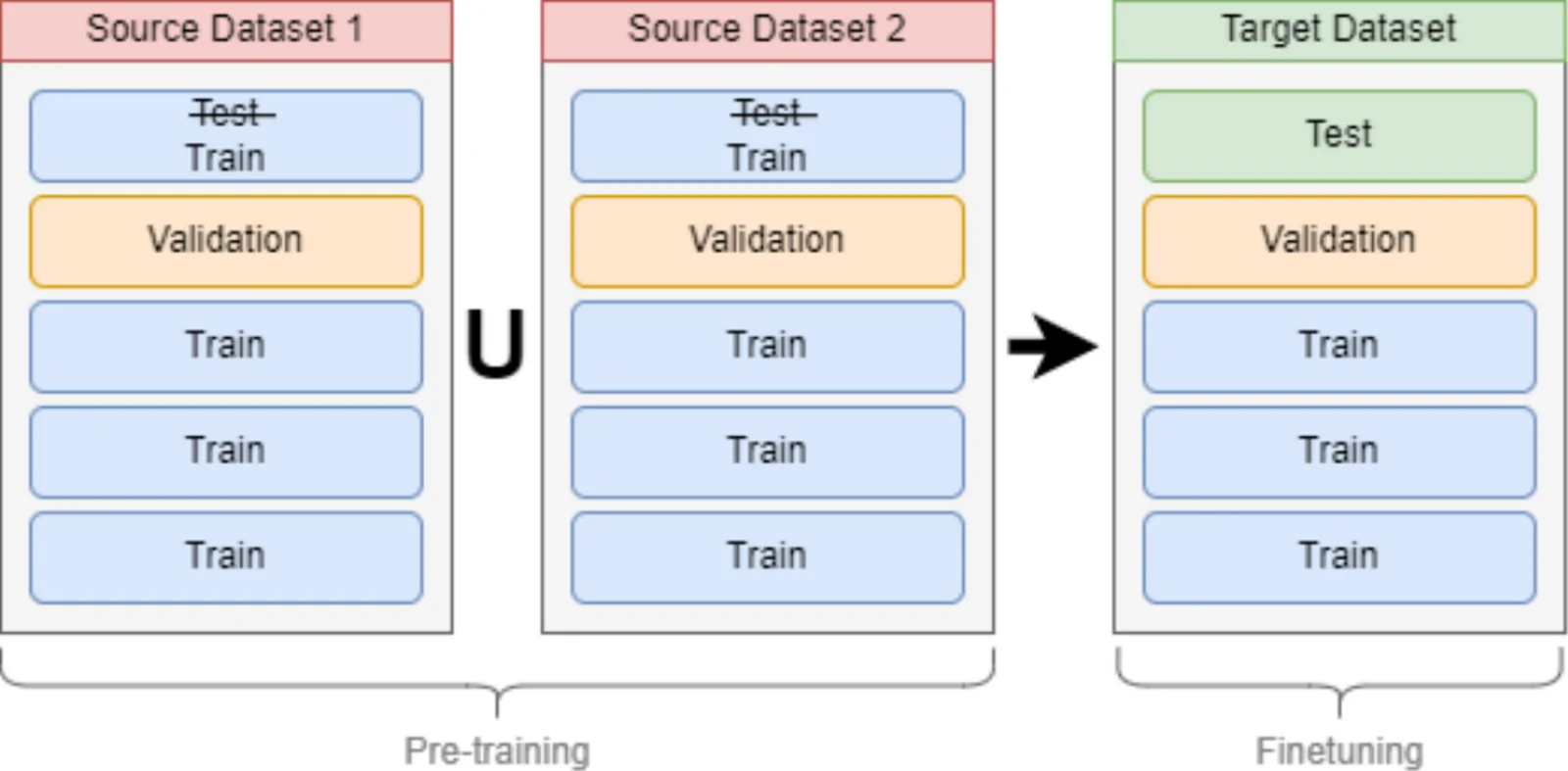
Illustration of our fivefold cross-validation strategy for transfer learning. The pre-training dataset is built as the union of the two source datasets. For each fold, test partitions of the pre-training dataset are reattributed to the training set. Cross-validation reduces the impact of random partitioning and ensures a more accurate evaluation of the model’s performance

Training occurred in two steps, namely, pretraining and finetuning. The combination of these two steps is what we refer to as transfer learning. Pretraining was done on two source datasets and finetuning on the third target dataset. For example, when Local A was the target dataset, PPMI and Local B were used as the source datasets for pretraining. This is represented in Fig. 5.

Training followed a common stratified fivefold train–validation–test scheme. Each dataset was randomly split into five non-overlapping partitions, each partition making up 20% of the dataset. Stratification makes partitions have the same prevalence as the dataset. Each fold contains 3 train partitions, 1 validation partition, and 1 test partition, yielding a 3:1:1 ratio. Using the union of both source datasets, train partitions were used to train models, while validation partitions were used to select the best model with the lowest validation loss over the training process. The test partition of the target dataset was used to compute performance metrics (detailed in Section "Evaluation Metrics"). For fold n°1, partition n°1 of the target dataset was used as the test partition, partition n°2 of both source datasets was used as the validation partition, and the remaining partitions for training. For fold n°2, testing was done with partition n°2, validation with partition n°3, and so forth. This approach was used to follow the principles of cross-validation, with the intent of estimating model performance in a more reliable way that depends less on the random shuffling and splitting of the data into train, validation, and test sets.

There were two key differences between pretraining and finetuning. First, during the pretraining phase, no test was necessary, so the test partition was added to the training data, as depicted by the strikethrough on the first row of partitions in Fig. 5. Second, k-folding was only applied to the finetuning step. Instead, only one model per target dataset was pretrained on the two source datasets using the first fold and served as the starting point of the finetuning of the five folds based on the target dataset. The main motivation was to reduce experimentation time. This was possible because there was no overlap between source and target datasets and therefore no leakage.

#### Augmentations

During both training steps, augmentations were used on the training set, each with an independent probability $\mathrm{p}$ of being applied:

1. DAT SPECT images: flip along any axis (*p* = 0.5); rotation by up to 20° along any axis (*p* = 0.8); translation by up to 2 voxels along any axis (*p* = 0.5); additive Gaussian noise centered on zero and with a standard deviation of 0.01 (*p* = 0.2).
2. Scalar data: additive zero-mean Gaussian noise with a standard deviation of 0.1 (*p* = 1.0); feature dropout (*p* = {0.4; 0.2; 0.05}). Feature dropout occurs in three stages, each with their own motivation. In all stages, when a feature is dropped, the availability vector is updated to reflect the fact that the feature is now missing. The first feature dropout stage (*p* = 0.4) aims at facilitating the transfer learning between the PPMI dataset and other datasets by dropping all features absent from the PPMI dataset (i.e., the 10 last striatal image features). The second stage aims at training the model to work with any set of features by giving each feature an independent probability (*p* = 0.2) to be dropped. The third stage further reinforces incomplete multimodal learning by dropping all scalar features at once (with *p* = 0.05).

### Experiments

#### Evaluation Metrics

The reported metrics are the area under the receiver operating characteristic curve (AUROC), the equal error rate (EER) computed from the ROC curve, the sensitivity, the specificity, and the accuracy. The EER is the point at which the false positive rate and the false negative rate are equal. Sensitivity, specificity, and accuracy are reported for the threshold of the EER. For other works, we reported values found in their original papers. When we compared our different models, statistical significance was assessed using a naïve k-fold cross-validation McNemar’s test with a significance α level at 0.05 [44]. This test combines the statistics of each fold and measures the statistical difference in accuracy at EER between two models.

#### Description of the Experiments

To start with, a full model evaluation is performed to compare it to the state of the art. For each dataset, we pretrained and finetuned our multimodal model as described in Section "Training and Testing". Once all models were trained, we computed the evaluation metrics on the corresponding test dataset.

Afterwards, we conducted additional experiments to perform an ablation study, where various components of our approach were disabled to assess their impact on the model’s performance:

1. No transfer learning. We trained new models on the target dataset, without pretraining, and then compared their performance.
2. Unimodal—image only. We assessed the contribution of scalar data in the multimodal setup by training new unimodal imaging models, which consisted only of the ViT of the original architecture (and a single classification layer). We also investigated the removal of the transfer learning component in this unimodal setup.
3. Unimodal—scalar data only. We assessed the relevance of image data in the multimodal setup. To do that, only using scalar data, we fit k unimodal logistic regression models, which were particularly well suited for this case.

#### Explainability

Using the multimodal models trained with transfer learning, we produced four explainability outputs to assess their relevance in clinical practice:

1. Fusion attention map: the attention rollout map produced by the model to fuse the different modalities [45]. Because the norm of the different input tokens highly depended on their related modality, we multiplied the attention rollout map by the norm of input tokens as proposed by Abnar and Zuidema [45].
2. Image attention map: the spatial attention rollout map produced by the image encoder [45].
3. Non-image saliency map: obtained through backpropagation of gradients, here it is produced for scalar data [46].
4. Image occlusion map: obtained by occluding parts of the image and measuring the absolute difference in the model prediction [47, 48].

## Results

Since we used a fivefold cross-validation, we reported the results of the experiments as “mean [CI],” where CI is the 95% confidence interval.

### Comparison with the State of the Art

Our full model achieved similar results to state-of-the-art methods also evaluated on the PPMI dataset (Table 3). In comparison to the PPMI dataset, our method performed slightly worse for the Local A dataset and reached similar performance for the Local B dataset.

**Table 3:** Performance of our approach on three datasets in %. On the PPMI dataset, our model achieved performance competitively with state-of-the-art methods [3–6, 49, 50]

Method	AUROC	EER	Sensitivity	Specificity	Accuracy
Ours (PPMI)	97.4 [92.5–100.0]	4.3 [0.0–10.5]	97.0 [91.4–100.0]	91.9 [77.5–100.0]	95.5 [89.4–99.3]
Ours (Local A)	92.6 [82.0–99.4]	14.4 [2.4–28.2]	84.4 [54.5–100.0]	79.9 [54.2–100.0]	82.1 [69.6–93.2]
Ours (Local B)	98.6 [94.5–100.0]	4.0 [0.0–11.4]	95.0 [88.9–100.0]	95.9 [82.4–100.0]	95.3 [88.7–99.1]
Choi et al. [6]	99.0	N/A	94.2	100.0	96.0
Kurmi et al. [3]	N/A	N/A	98.8	97.7	98.5
Magesh et al. [50]	94.0	N/A	97.5	90.9	95.2
Ortiz et al. [5]	95.0	N/A	96.0	94.0	95.0
Pianpanit et al. [49]	98.7	N/A	96.4	97.9	96.9
Quan et al. [4]	99.1	N/A	98.9	97.7	98.5

### Ablation Studies

For both local datasets, the contribution of multimodality and transfer learning ranged from statistically non-significant to significant performance improvements. For the PPMI dataset, we observed a statistically non-significant decrease in performance due to multimodality and no effect from transfer learning. The smallest impact was observed for the PPMI dataset (Table 4) and the highest for the Local A dataset (Table 5). On the Local B dataset, the impact was in between, except when comparing with scalar-only models, which performed similarly (Table 6).

**Table 4:** Performance of our approach across multiple ablation studies on the PPMI dataset in %. No methods were found to be statistically different on this dataset

Transfer	Modalities	AUROC	EER	Sensitivity	Specificity	Accuracy
Yes	All	97.4 [92.5–100.0]	4.3 [0.0–10.5]	97.0 [91.4–100.0]	91.9 [77.5–100.0]	95.5 [89.4–99.3]
No	All	97.7 [93.2–100.0]	4.6 [0.5–10.4]	96.6 [91.4–100.0]	93.0 [77.5–100.0]	95.5 [91.7–98.5]
Yes	Image	98.5 [95.1–100.0]	4.6 [0.0–11.2]	96.1 [90.4–100.0]	94.9 [84.6–100.0]	95.8 [90.9–99.2]
No	Image	98.5 [94.5–100.0]	4.1 [0.5–8.9]	96.3 [91.4–100.0]	95.4 [85.0–100.0]	96.1 [91.7–99.3]
N/A	Scalars	98.2 [95.6–99.9]	4.5 [0.5–10.0]	95.3 [89.2–100.0]	93.9 [77.5–100.0]	94.9 [90.2–98.5]

**Table 5:** Performance of our approach across multiple ablation studies on the Local A dataset in %. All methods using images were found to be statistically better than the scalar-only method on this dataset

Transfer	Modalities	AUROC	EER	Sensitivity	Specificity	Accuracy
Yes	All	92.6 [82.0–99.4]	14.4 [2.4–28.2]	84.4 [54.5–100.0]	79.9 [54.2–100.0]	82.1 [69.6–93.2]
No	All	92.3 [81.6–99.6]	13.9 [2.1–29.5]	82.6 [45.5–100.0]	81.6 [50.0–100.0]	82.1 [67.4–93.5]
Yes	Image	93.0 [82.2–99.6]	13.3 [2.1–28.2]	83.4 [52.4–100.0]	87.5 [62.5–100.0]	85.5 [71.7–95.7]
No	Image	91.1 [77.5–99.6]	16.0 [2.3–31.9]	84.3 [57.1–100.0]	82.4 [58.3–100.0]	83.4 [69.6–95.7]
N/A	Scalars	92.4 [80.7–100.0]	13.1 [0.0–28.2]	83.5 [45.5–100.0]	83.2 [50.0–100.0]	83.4 [67.4–100.0]

**Table 6:** Performance of our approach across multiple ablation studies on the Local B dataset in %. The multimodal method with no pretraining was found to be statistically better than the unimodal image method with pretraining on this dataset

Transfer	Modalities	AUROC	EER	Sensitivity	Specificity	Accuracy
Yes	All	98.6 [94.5–100.0]	4.0 [0.0–11.4]	95.0 [88.9–100.0]	95.9 [82.4–100.0]	95.3 [88.7–99.1]
No	All	98.7 [95.1–100.0]	4.4 [0.0–11.4]	96.7 [88.9–100.0]	77.1 [0.0–100.0]	90.4 [67.9–99.1]
Yes	Image	97.5 [93.5–99.8]	6.8 [0.7–13.6]	93.3 [86.1–98.6]	90.7 [71.4–100.0]	92.5 [85.0–98.1]
No	Image	97.2 [92.4–99.6]	7.4 [2.2–14.1]	92.2 [84.7–98.6]	89.5 [71.4–100.0]	91.3 [85.0–96.2]
N/A	Scalars	98.3 [93.9–100.0]	3.5 [0.0–10.8]	96.1 [88.9–100.0]	95.9 [82.4–100.0]	96.0 [90.5–100.0]

The performance of scalar-only models highly depended on the dataset. These models achieved similar performance as other methods on PPMI (Table 4) and the Local B (Table 6) datasets and statistically lower performance than image methods on the Local A (Table 5) dataset.

**Fig. 6:**
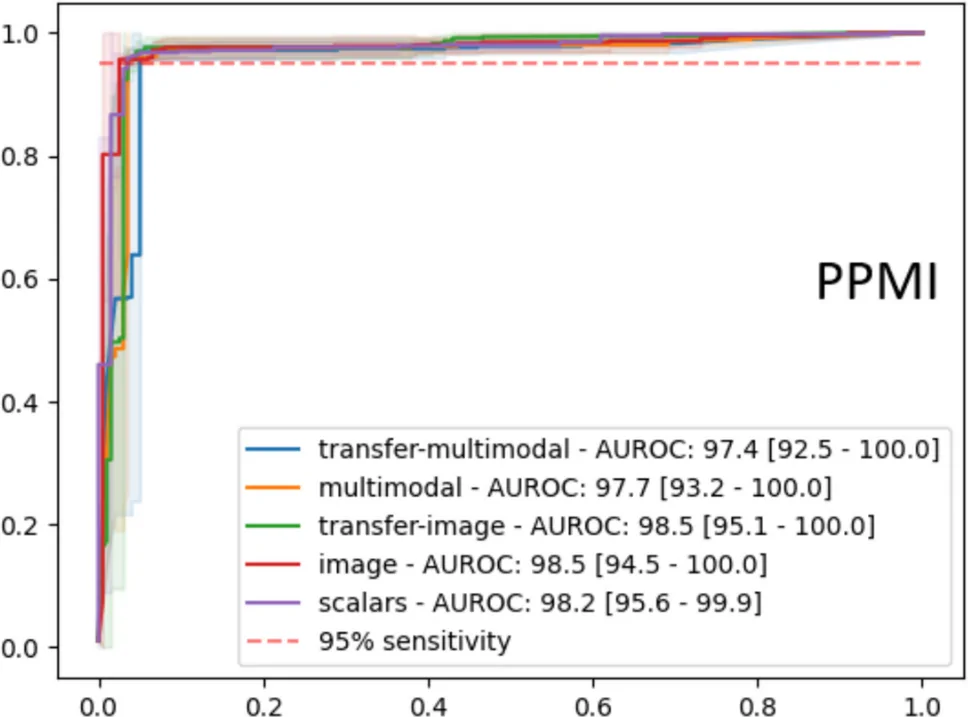
ROC curves for the PPMI dataset. Plain lines represent the mean ROC curves while the semi-transparent areas represent the uncertainty related to k-folding of the cross-validation. The dashed line represents the 95% sensitivity threshold

**Fig. 7:**
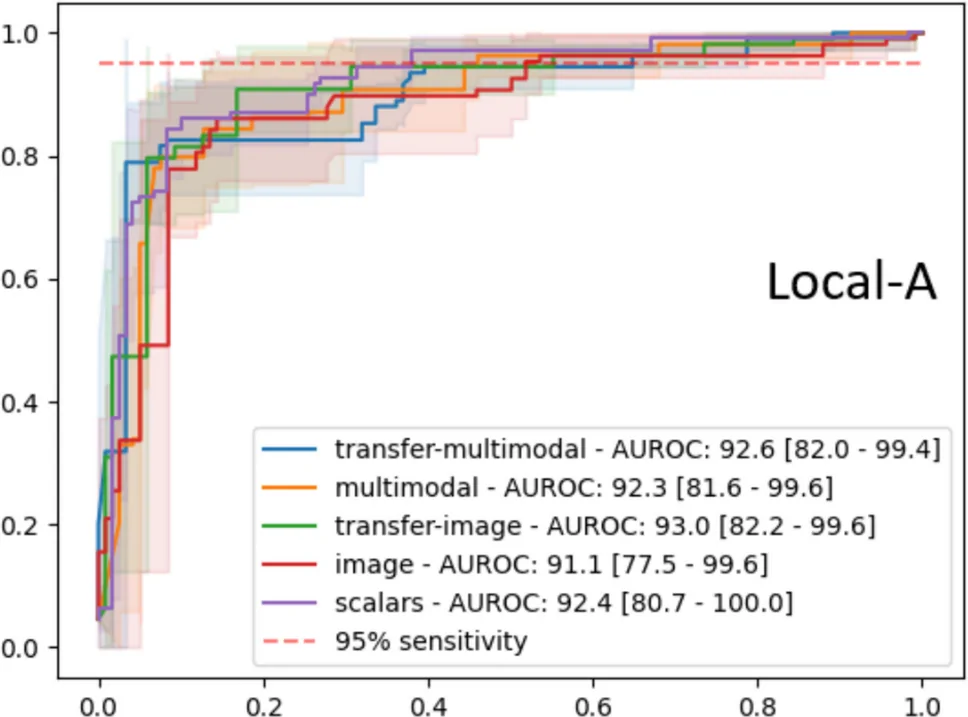
ROC curves for the Local A dataset. Plain lines represent the mean ROC curves while the semi-transparent areas represent the uncertainty related to k-folding of the cross-validation. The dashed line represents the 95% sensitivity threshold

**Fig. 8:**
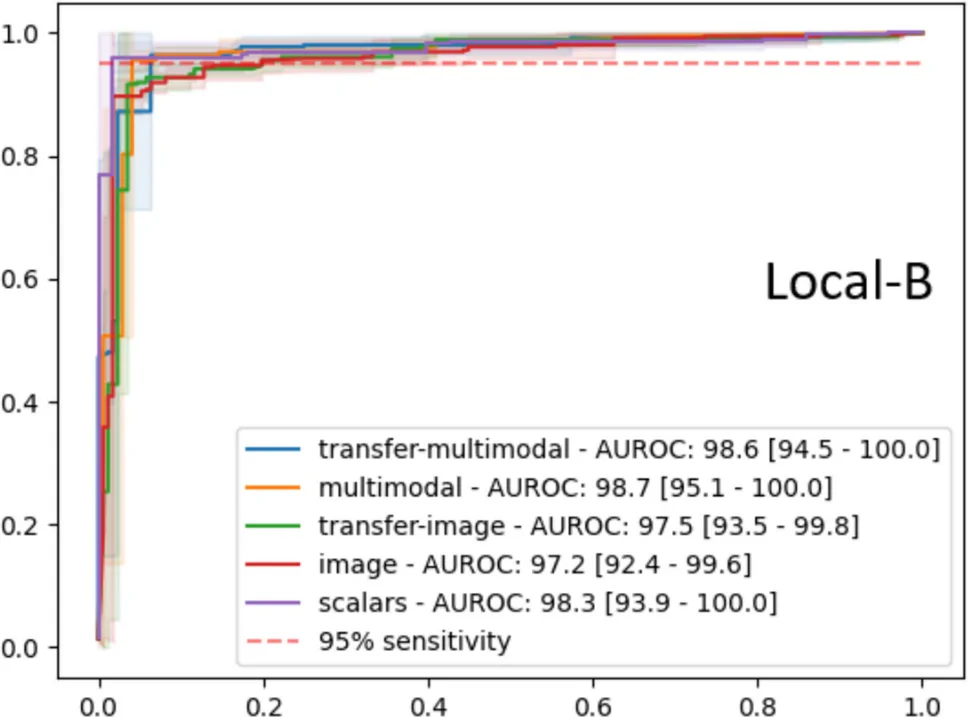
ROC curves for the Local B dataset. Plain lines represent the mean ROC curves while the semi-transparent areas represent the uncertainty related to k-folding of the cross-validation. The dashed line represents the 95% sensitivity threshold

We plotted the ROC curves for each ablation study for the PPMI (Fig. 6), Local A (Fig. 7), and Local B (Fig. 8) datasets. Due to k-folding, we had five ROC curves per approach and per dataset. These five ROC curves were aggregated using the mean, represented by a solid line, and the standard deviation, represented by a semi-transparent area.

### Explainability

#### Multimodal Fusion Attention Weights

**Fig. 9:**
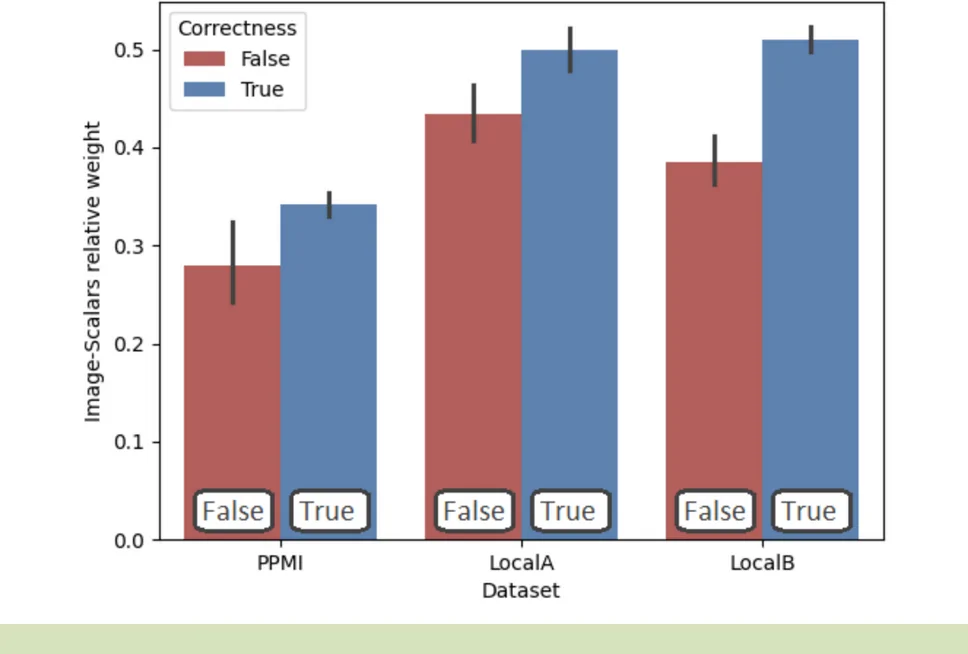
Image-scalar relative attention per dataset and prediction correctness. Higher values indicate higher attention to images

Using rollout attention [45], we were able to compute how much attention was given to each modality by the model to produce the fused representation. Given the resulting image and scalar attention weights ${w}_{image}$ and ${w}_{scalar}$ used by the fusion transformer to merge the different modalities, we computed the image-scalar relative attention ${w}_{image}/({w}_{image}+{w}_{scalar})$, depicted in Fig. 9. In average, models gave more attention to scalars than image information. However, models gave consistently less attention to images when they made incorrect predictions.

#### Scalar Data Saliency

**Fig. 10:**
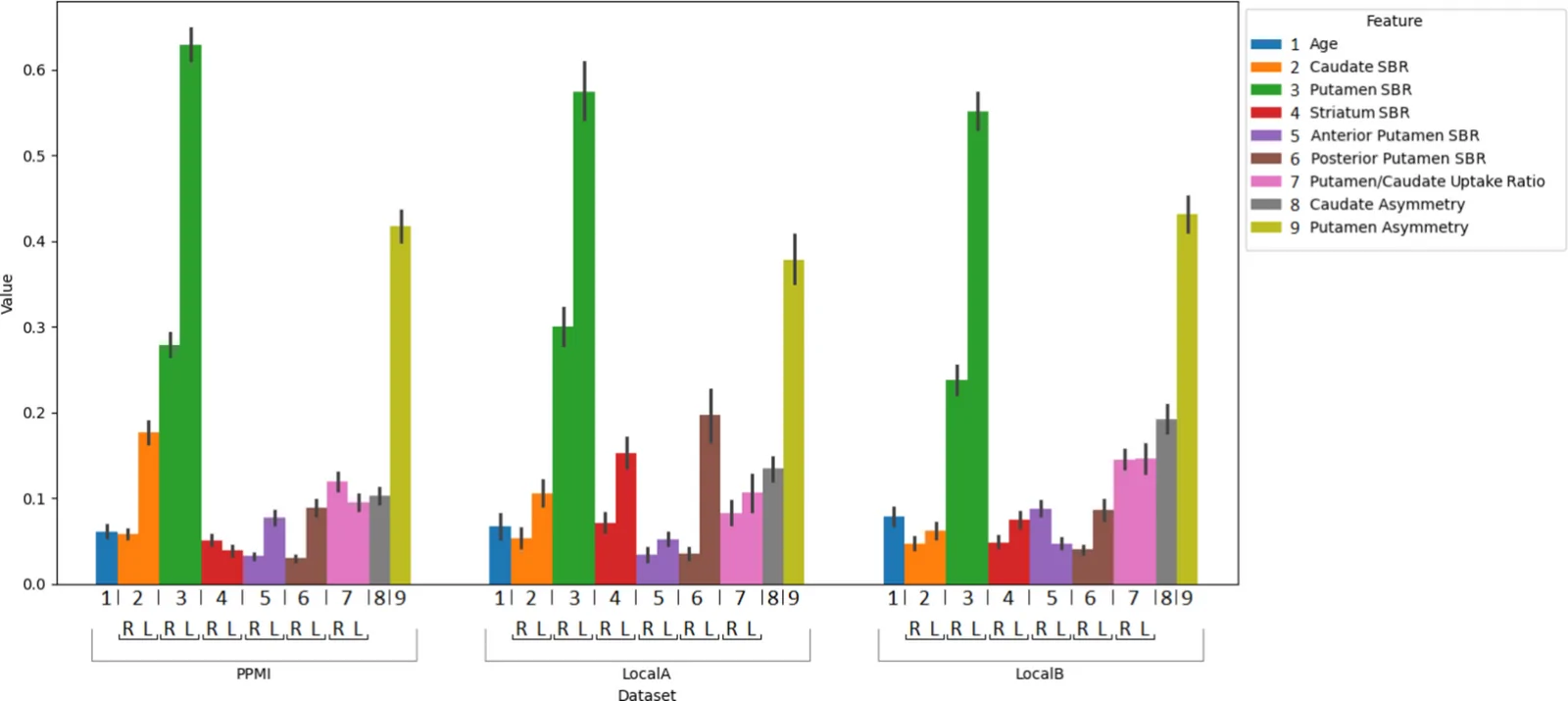
Scalar data saliency for each dataset, modeling the weight of the feature in the model prediction. When applicable, right/left striatal image features are grouped and share the same color. Higher values can be interpreted as higher influence on the model’s prediction

Using saliency maps [46], we were able to estimate the average saliency value for each scalar feature. A feature with a higher saliency value can be interpreted as a feature that would have a higher impact on the prediction of the model for a given alteration. In Fig. 10, we show the average saliency value for each scalar feature, separated by dataset. We can notice that the SBR for the left putamen was the most used feature overall. It is followed closely by the putamen asymmetry, even for the PPMI dataset for which this value was not available, probably due to the transfer learning from the local datasets. We also note that, overall, age did not seem to be a salient feature.

#### Spatial Attention and Occlusion Maps

**Fig. 11:**
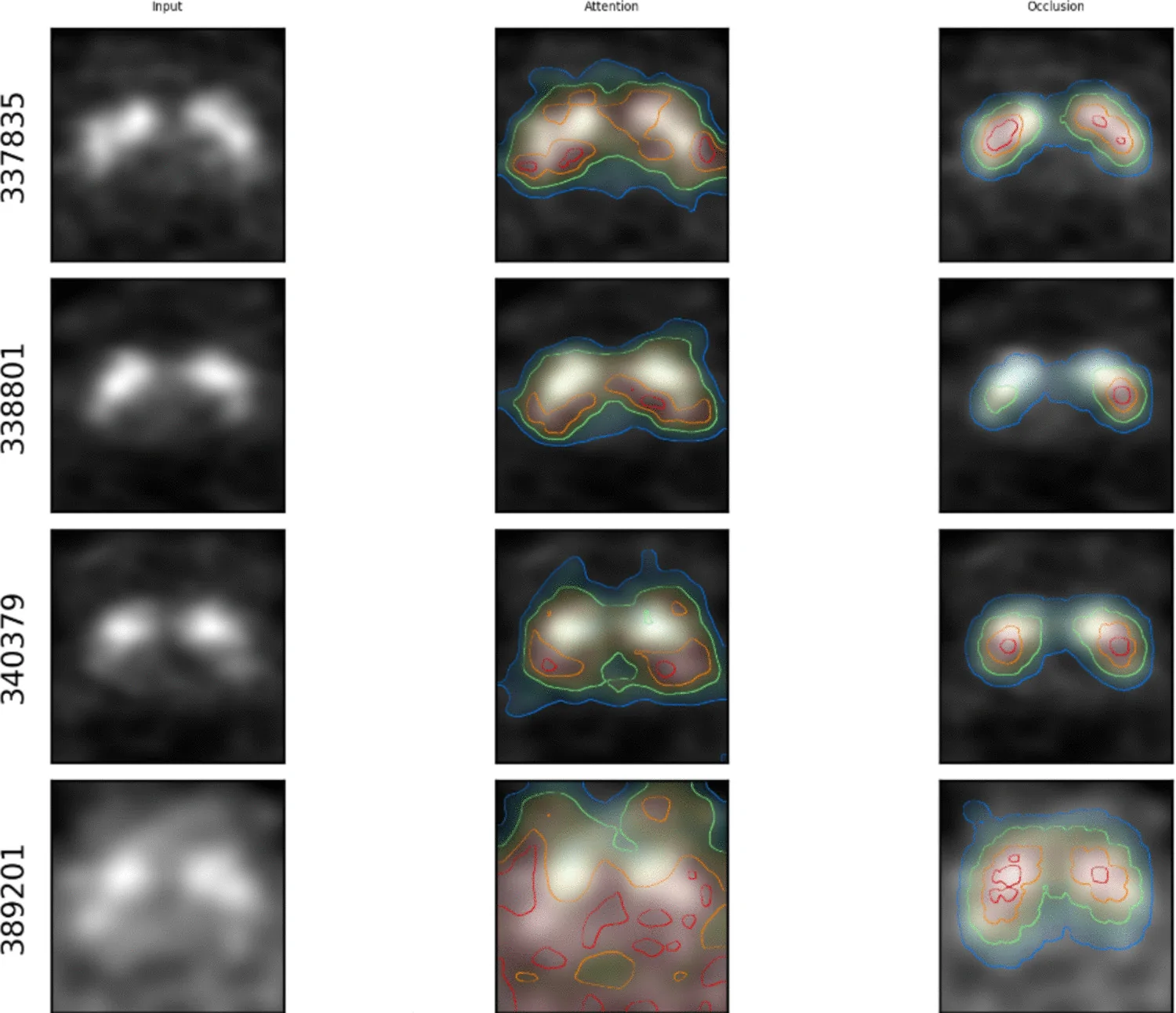
Different cases of visual explainability outputs for the PPMI dataset, using the spatial attention and occlusion visualization techniques. In general, the model’s focus is adequately on the striatum area, especially in the putamen regions. Case 389,201 shows attention maps may be less robust than occlusion maps on degraded image quality and that both methods are limited by image quality

The use of a ViT as the image encoder allowed the computation of spatial attention maps. As with fusion weights in Section "Multimodal Fusion Attention Weights", we used the rollout attention to produce these attention maps. We also computed spatial occlusion maps where a sliding window occludes one region of the image at a time, measures the absolute shift in the model’s prediction, and attributes it to the occluded region. Figure 11 shows a few examples of these spatial maps using cases from the PPMI dataset. These visualizations highlight that models consistently focus on the striatum, especially in the putamen regions that are commonly scrutinized by human readers. Figure 11 also shows the respective resilience of these approaches to noisy inputs, especially for the occlusion visualization technique.

### Edge Case Analysis

We identified three edge cases in the Local A dataset using our best model. Two involved discrepancies between the clinician’s initial visual interpretation and the confirmed clinical diagnosis. The third edge case corresponded to the sample with the largest prediction error made by the model.

**Fig. 12:**
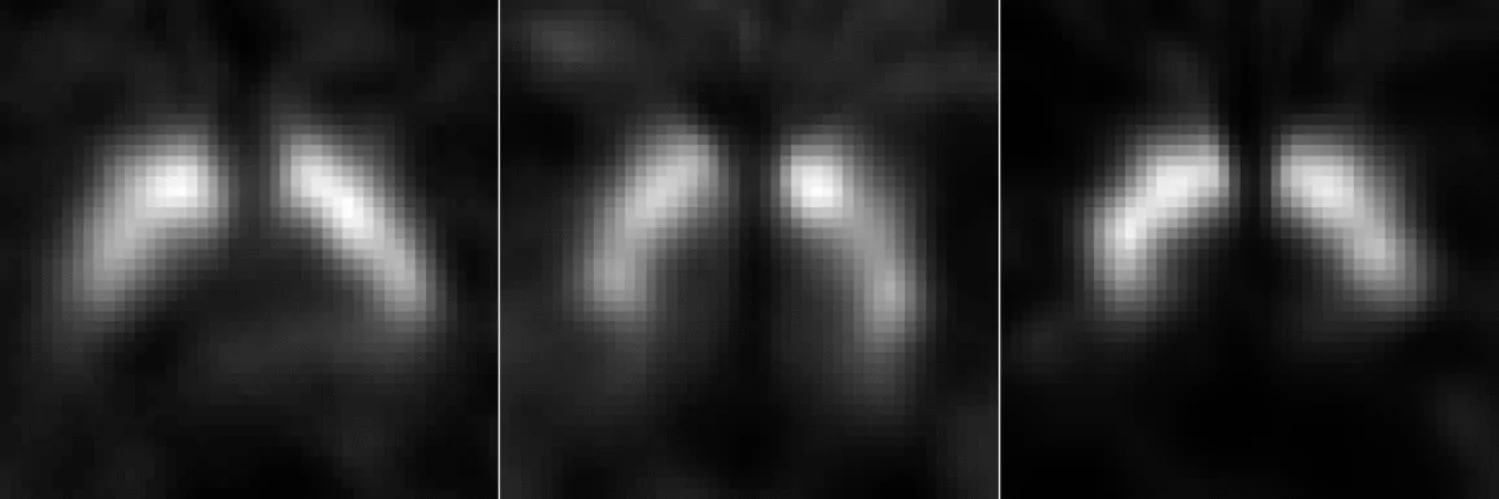
Edge cases from the Local A dataset using our best model. Left: degenerative case with normal SBR values visually interpreted as negative, but correctly classified by the AI model. Center: non-degenerative case visually interpreted as positive and also misclassified by the model. Right: degenerative case with normal SBR values, misclassified as control by the AI model, while the clinician relied on an asymmetry in the left putamen to flag it as positive

For the left case in Fig. 12, the model correctly classified a sample initially interpreted as negative by the clinician, with all SBR values within the normal range, later confirmed as degenerative—highlighting the potential of our approach to reduce false negatives. For the center case, although misclassified as degenerative, the model probability (40.4%) was close to the EER threshold (34.0%) and might have been discarded in practice due to this proximity. For the right case, the model misclassified this image as control. All SBR values were within the normal range, but the clinician was able to correctly interpret the case as positive thanks to a visual asymmetry in disfavor of the left putamen.

## Discussion

To ensure that our method was competitive with state-of-the-art methods, we ran experiments on the PPMI dataset. This showed that our method, while seemingly not achieving new best performance on this dataset (which already stood at 99.1% AUROC), was in the expected range for competitiveness. However, contrary to the previous work we are comparing our method to, we used k-fold cross-validation with non-overlapping fold partitions to strengthen the reliability of our results. In fact, the absence of cross-validation in previous studies may have artificially inflated performance due to a favorable random split between training and testing datasets. For example, for fold n°2 on the PPMI dataset, our model yielded the highest AUROC value of 99.1%, similarly to the works of Choi et al. [6] and Quan et al. [4]. The goal was to ensure competitiveness while participating in the establishment of a k-fold cross-validated baseline and while working in a constrained setup where information can be partial or missing.

Once this baseline comparison on the PPMI dataset was made, we were able to evaluate our method on our local datasets. We needed to make sure that our method did not only work on the highly curated PPMI dataset, but that it was also suitable for clinical datasets collected in varying conditions. This showed our method could achieve the same high performance on a local dataset, namely, the Local B dataset, but that there was variability depending on the dataset. In fact, while performance was still high on the Local A dataset, it was lower than on the PPMI and Local B datasets. We identified several potential causes for this. The Local A dataset is about 55%–65% smaller than the two other datasets and its prevalence is about 20%–25% lower. Additionally, the camera for the Local A dataset was not specifically built for the head, yielding a subjectively worse image quality. However, this estimation was only subjective, as assessing the objective image quality difference between datasets was not done. To our best knowledge, there are no objective metrics to specifically assess the quality of DAT images, and it is a work in progress we are conducting.

Having established that our method performed well on both the PPMI dataset and our local datasets, we assessed the contribution of transfer learning and multimodality management. Transfer learning showed it only had a significant impact on the Local A dataset, for example, with an increase of the AUROC by an average of + 1.1% (+ 0.3% in the multimodal setup and + 1.9% in the unimodal setup). This was consistent with expectations, as the Local A dataset was significantly smaller than the other two. However, we were not able to determine whether there was an absolute dataset size above which transfer learning seemingly ceased to improve performance, or if the source dataset had to be relatively bigger than the target dataset. Regarding our multimodal architecture, it had a positive impact on our local datasets for which multimodal models consistently matched the performance of the best unimodal approach. Conversely, multimodal models performed slightly worse than unimodal models on the PPMI dataset. This suggests our multimodal models could leverage the best available modality, reducing the need to determine which modality would be optimal. However, on the PPMI dataset, these multimodal models may have been hindered by the partial nature of scalar data as 10 striatal features were missing. While our method was still able to perform well in this context where inputs were partial, future work should investigate how to better retain performance observed in unimodal models. The use of self-supervised learning (SSL) can be an interesting approach for pretraining for datasets with limited annotations [51]. Indeed, SSL facilitates the extraction of intrinsic data patterns [52, 53] and, in a multimodal context, could help models uncover new relationships between modalities and clinical diagnoses.

We then turned our attention to the analysis of the explainability tools allowed by our methods. We extracted the fusion transformer’s attention and looked at how much attention was given to images versus scalar data. This showed that, while attention was generally evenly allocated between images and scalar data, prediction errors were more frequent when images were given less attention. This suggests that DAT SPECT images carried critical information that was missing from age and striatal image features. This further reinforces the idea that striatal image features may not always be enough to reach a decisive diagnosis. Reduced image attention could also reflect limitations in either image quality or interpretability. In future work, clinicians could be given the possibility to alter attention weights, to either increase or decrease attention to images, based on their reading of the image and their experience. Such investigation should measure the impact of such human intervention in the model’s performance and the impact for the clinician, both in terms of usability and trust. The three other explainability tools focused more on analyzing the region of the striatum. Occlusion maps (Fig. 11) highlighted that the model paid high attention on the putamen regions, which is a reassuring and adequate focus since DAT imaging findings are considered abnormal with unilateral or bilateral loss of striatal uptake, generally observed earlier in the putamen [54]. Moreover, the saliency analysis on the striatal image features (Fig. 10) highlighted a left putamen focus which may indicate that the model is also considering the laterality as a relevant clinical factor. This is consistent with the hypothesis that DAT imaging in the premotor phase of PD would first show detectable abnormalities unilaterally [55], especially on the left hemisphere due to right-handiness predominance [56].

Regarding “edge cases,” their analysis helped us to better understand some weaknesses of the proposed model. Two of the three edge cases revealed to be challenging to interpret visually, as the initial clinician’s visual interpretation differed from the confirmed diagnosis. This may indicate that the proposed model is better suited to assist the clinician on simpler cases to allow the clinician to allocate more time on more complex cases. To improve on this, future work should investigate ways for the model to score its confidence about its prediction, making complex cases easier to identify. Ideally, the model would identify cases where it would have an even chance of making the right or the wrong prediction.

Besides future work to address the mentioned limitations, one area we could not explore was the model’s prognosis capabilities as our datasets did not provide the necessary information. However, future research is needed on this clinically relevant feature as reported by previous work [57–59].

## Conclusions

This study investigated the potential of multimodal modeling and transfer learning to detect positive scans using DAT SPECT images and scalar data. Our approach demonstrated improved versatility and performance, particularly in more challenging contexts where datasets can be smaller or have subjectively lower quality data with higher variability as often found in clinical practice. Put otherwise, our method allows for gathering less data, on a broader range of SPECT scanners, while retaining performance similar to more ideal cases such as the PPMI dataset. The work presented also included a few explainability methods, covering the key components of our models. These explainability methods helped gain insight into our models, to allow for better understanding and future improvements. More importantly, by displaying elements such as the attention of the model at different stages, these explainability methods should help build trust with clinicians, thus facilitating adoption of advanced imaging methods in clinical practice.

## Data Availability

PPMI data is available at http://www.ppmi-info.org/access-data-specimens/download-data. Local datasets are not available due to restrictions from the Geneva Canton Ethical Committee.
